# α-Tocopherol succinate enhances pterostilbene anti-tumor activity in human breast cancer cells *in vivo* and *in vitro*

**DOI:** 10.18632/oncotarget.23390

**Published:** 2017-12-17

**Authors:** Ka-Wai Tam, Chi-Tang Ho, Shih-Hsin Tu, Wen-Jui Lee, Ching-Shui Huang, Ching-Shyang Chen, Chih-Hsiung Wu, Chia-Hwa Lee, Yuan-Soon Ho

**Affiliations:** ^1^ Graduate Institute of Clinical Medicine, College of Medicine, Taipei Medical University, Taipei, Taiwan; ^2^ Department of Surgery, Division of General Surgery, Shuang Ho Hospital, Taipei Medical University, New Taipei City, Taiwan; ^3^ Department of Surgery, School of Medicine, College of Medicine, Taipei Medical University, Taipei, Taiwan; ^4^ Department of Food Science, Rutgers University, New Brunswick, NJ, USA; ^5^ Breast Medical Center, Taipei Medical University Hospital, Taipei, Taiwan; ^6^ Taipei Cancer Center, Taipei Medical University, Taipei, Taiwan; ^7^ Comprehensive Cancer Center of Taipei Medical University, Taipei, Taiwan; ^8^ Department of Neuroscience, College of Medical Science and Technology, Taipei Medical University, Taipei, Taiwan; ^9^ Department of Surgery, Division of Breast Surgery, Cathay General Hospital, Taipei, Taiwan; ^10^ Comprehensive Breast Health Center, Taipei Medical University Hospital, Taipei, Taiwan; ^11^ Department of Surgery, Mennonite Christian Hospital, Hualien, Taiwan; ^12^ Department of Surgery, En Chu Kong Hospital, New Taipei City, Taiwan; ^13^ Department of Laboratory Medicine, Shuang Ho Hospital, Taipei Medical University, Taipei, Taiwan; ^14^ School of Medical Laboratory Science and Biotechnology, College of Medical Science and Technology, Taipei Medical University, Taipei, Taiwan; ^15^ Ph.D. Program in Medicine Biotechnology, College of Medicine, Taipei Medical University, Taipei, Taiwan; ^16^ Department of Laboratory Medicine, Taipei Medical University Hospital, Taipei, Taiwan; ^17^ Graduate Institute of Medical Sciences, College of Medicine, Taipei Medical University, Taipei, Taiwan

**Keywords:** vitamin E, tocopherol-associated protein, α-tocopheryl succinate, pterostilbene, breast cancer

## Abstract

Vitamin E (Vit. E) is considered an essential dietary nutrient for humans and animals. An enormous body of evidence indicates the biological and protective effects of Vit. E consumption. Tocopherol-associated protein (TAP) is a major tocopherol-binding protein affecting Vit. E stimulation and downstream signaling transduction. However, how Vit. E utilizes TAP as an anti-cancer mechanism remains unclear. Microarray analysis of signature gene profiles in breast cancer cells treated with α-tocopheryl succinate (α-TOS, a Vit. E isoform) resulted in cell cycle arrest and anti-cancer activity in breast cancer cells. Pterostilbene (PS), a natural dietary antioxidant found in blueberries, in combination with α-TOS synergistically maximized breast cancer cell growth inhibition by disrupting signal transduction, transcription factors and cell cycle proteins. In a xenograft mouse model, PS treatment with Vit. E inhibited breast tumor growth and cell invasion, which were evaluated using our recently developed circulating tumor cell (CTC) detection assay. Because dietary Vit. E and PS supplementation contributed to preventative and therapeutic effects *in vitro* and *in vivo*, this combination may benefit breast cancer therapy in the clinic.

## INTRODUCTION

**Figure 1:**
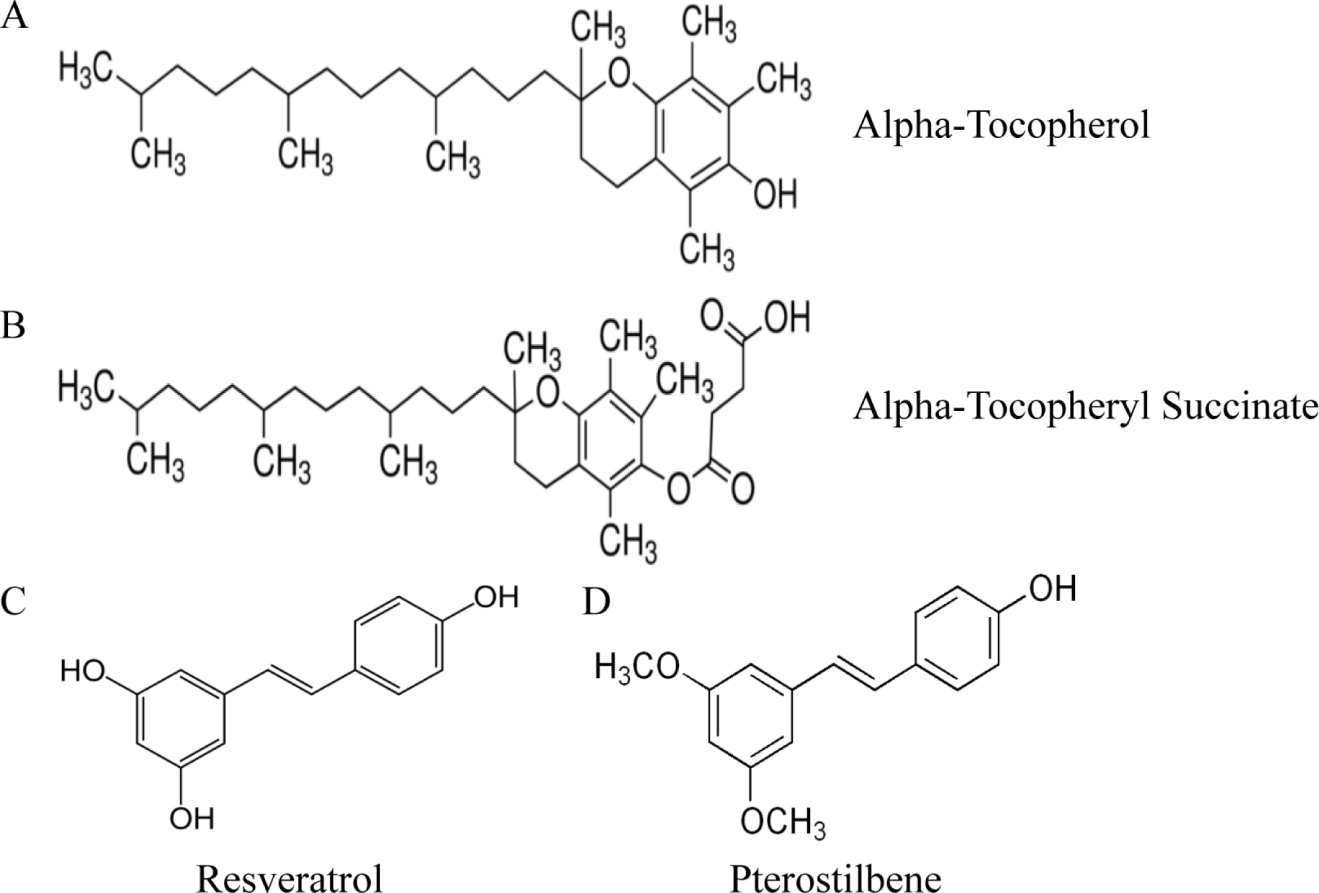
(**A**–**D**) Structures of pterostilbene, resveratrol, alpha-tocopherol, and alpha-tocopheryl succinate.

Vitamin E (Vit. E) is an important component for both cellular membranes and lipoproteins, and numerous evidences have demonstrated the effects of Vit. E in breast cancer prevention [1]. Among Vit. E isoforms, α-tocopherol (α-TP) is the main source and most active component (Figure 1A). In fact, the α-TP-derived compound found in green barley leaf extract α-tocopherol succinate (α-TOS, Figure 1B) has a higher anti-cancer effect than α-TP and others. α-TOS causes cell death mainly through the apoptotic pathway in human breast, neuroblastoma [2, 3], and prostate cancer cells [4]. In addition, α-TOS does not affect cell growth in most normal cells [5], making it an excellent candidate for dietary supplementation during cancer treatment. As a cellular binding protein for α-TP and α-TOS, tocopherol-associated protein (TAP) is believed to play an important molecular role in anti-cancer mechanisms in prostate, breast, liver, and brain tissues [6] but is nearly undetectable in most human tissues [7]. Furthermore, we previously showed that TAP was consistently preferentially expressed in normal breast tissue rather than in tumor lesions [8]. These studies indicate that using α-TP or its analogs (e.g., α-TOS) as dietary supplements may be beneficial during chemotherapeutic protocols for patients with breast cancer via a TAP activation mechanism.

Resveratrol (3,5,4′-trihydroxystilbene, Res; Figure 1C) is a well-known derivative of stilbene in red wine that can function as an anti-cancer agent [9–11]. As a natural dimethylated analog of Res, pterostilbene (3,5-dimethoxi-49-hydroxystilbene, PS; Figure 1D) has been found in various plants, including grapes, blueberries, and narra leaves [12]. Compared to Res, PS has a higher oral bioavailability (20% versus 80%), a higher potential for cellular uptake, a longer half-life (14 minutes versus 105 minutes) [13, 14], and more potent anti-tumor activity [15]. A synergistic effect of natural antioxidants (Res or PS) and Vit. E was proposed in a previous study [16], as the antioxidant action of Res involved trapping the propagating lipid peroxyl radical and reducing the α-TP radical to regenerate α-TP and enhance its antioxidant efficiency. The result implies that synergism between Res and α-TP derivatives during cancer therapy eliminates excessive oxidative damage and helps alleviate adverse effects.

Our previous study showed that TAP may act as a tumor-suppressive protein in breast cancer development [8]. A follow-up question would be whether Vit. E-induced TAP activation could be used in breast cancer therapy, especially for triple negative breast cancers (TNBC). In the current study, we aimed to uncover the anti-cancer mechanisms of α-TOS exposure through the TAP protein in TNBC cells by analyzing cell proliferation, signal transduction and cell cycle alterations. In addition, the synergistic anti-cancer effect of a natural compound in combination with α-TOS treatment was evaluated *in vitro* and *in vivo*. Our recently developed circulating tumor cell (CTC) detection assay was used on a xenograft-bearing animal model to determine whether Vit. E and PS exposure could suppress both tumor growth and metastasis [17]. These data help elucidate whether the use of both α-TP or its analogs and PS as supplements may be beneficial during cancer therapy for the treatment of human breast cancers.

## RESULTS

### Gene expression microarray profiling of breast cancer cell regulation via α-TOS exposure

**Figure 2:**
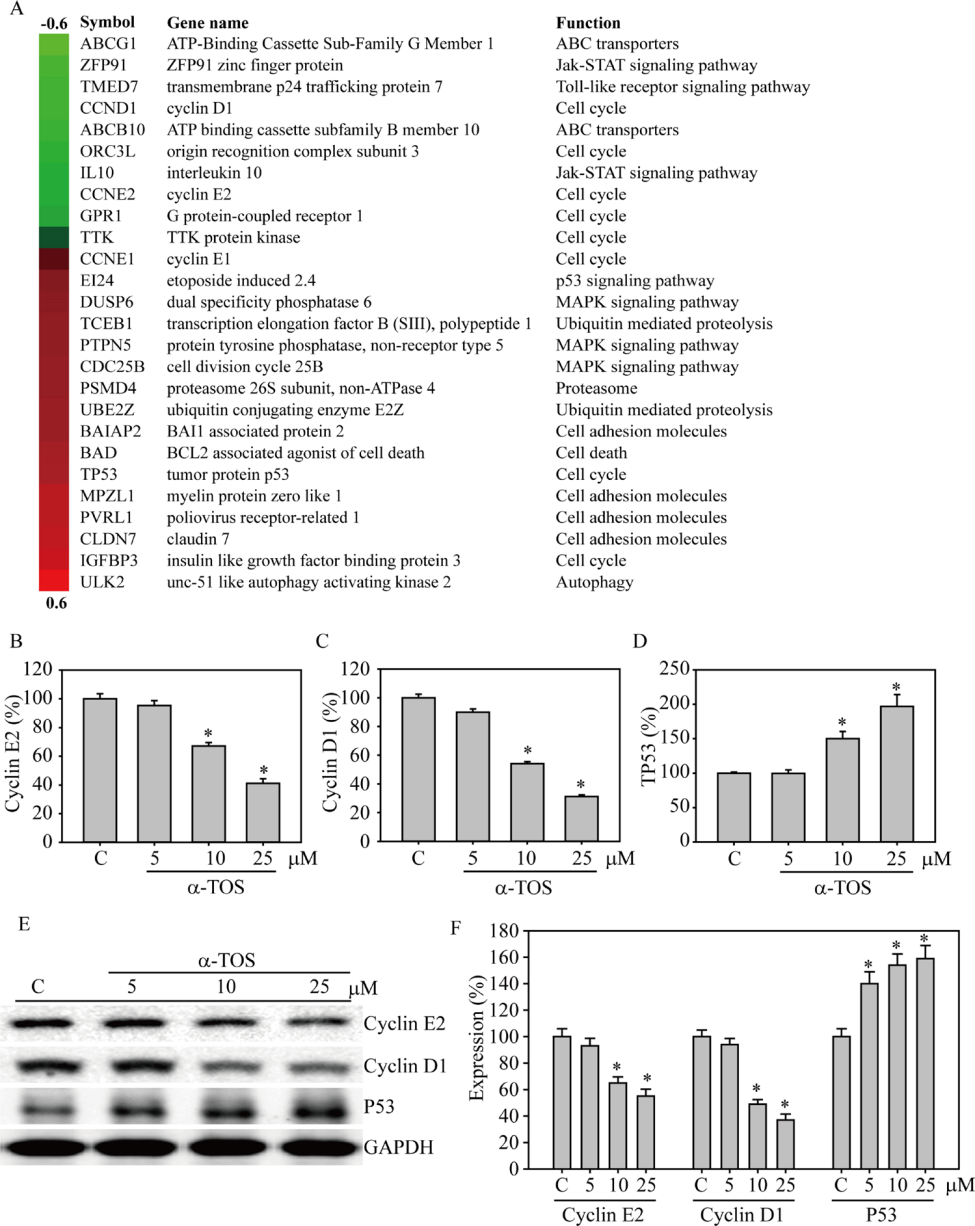
DAVID functional annotation cluster analysis of normalized and annotated genes during α-TOS treatment in breast cancer cells (**A**) The green and red colors represent microarray analysis with or without 10 μM α-TOS treatment in MDA-MB-231 breast cancer cells for 24 hours. Genes were selected and categorized by DAVID functional annotation cluster analysis and listed by their symbol, name, and function. Q-PCR analysis of (**B**) cyclin E2, (**C**) cyclin D1, and (**D**) P53 gene expression changes in MDA-MB-231 breast cancer cells treated with 0–25 μM α-TOS for 24 hours. (**E**) Western blot and (**F**) densitometry analyses of cyclin E2, cyclin D1, and P53 protein expression after 24 hours of 0–25 μM α-TOS treatment in MDA-MB-231 breast cancer cells. All statistical tests were two-sided and compared to control. *P*-values less than 0.05 are indicated with an asterisk.

A Database for Annotation, Visualization and Integrated Discovery (DAVID) heat map identified the top 26 genes that were up- and down-regulated by α-TOS exposure and functionally clustered into common gene ontology (GO) terms (Figure 2A, Supplementary Table 2). The clustered genes were related to cellular movement or metastasis [18], cell cycle regulation, signaling transduction [19], death/apoptosis, and growth [20]. Among the cell cycle regulation genes, both *CCND1* and *CCNE2* were significantly reduced, whereas cell cycle suppressive genes, such as *EI24*, *TP53*, and *IGFBP3*, were increased during α-TOS exposure. Meanwhile, cell adhesion-associated genes, such as *MPZL1*, *PVRL1*, and *CLDN7*, were also elevated by α-TOS exposure, indicating their decreased cancer metastasis ability.

To confirm the microarray data, α-TOS exposure was increased from 0 to 25 μM, which significantly decreased cyclin E2 and cyclin D1 gene expression (*p* < 0.05) (Figure 2B and 2C). In addition, Figure 2D shows that P53 gene expression was dramatically enhanced 1.5- and 2-fold with 10 and 25 μM α-TOS treatment, respectively (*p* < 0.05). The protein expression levels of cyclin E2 and cyclin D1 were significantly decreased by α-TOS exposure in a dose-dependent manner, with a maximum protein suppression of 58% and 39%, respectively, following exposure to 25 μM of α-TOS (*p* < 0.05), whereas P53 protein expression was significantly increased by 160% after 25 μM α-TOS exposure (*p* < 0.05), as determined by western blotting (Figure 2E and 2F).

### TAP-mediated cell cycle regulation

**Figure 3:**
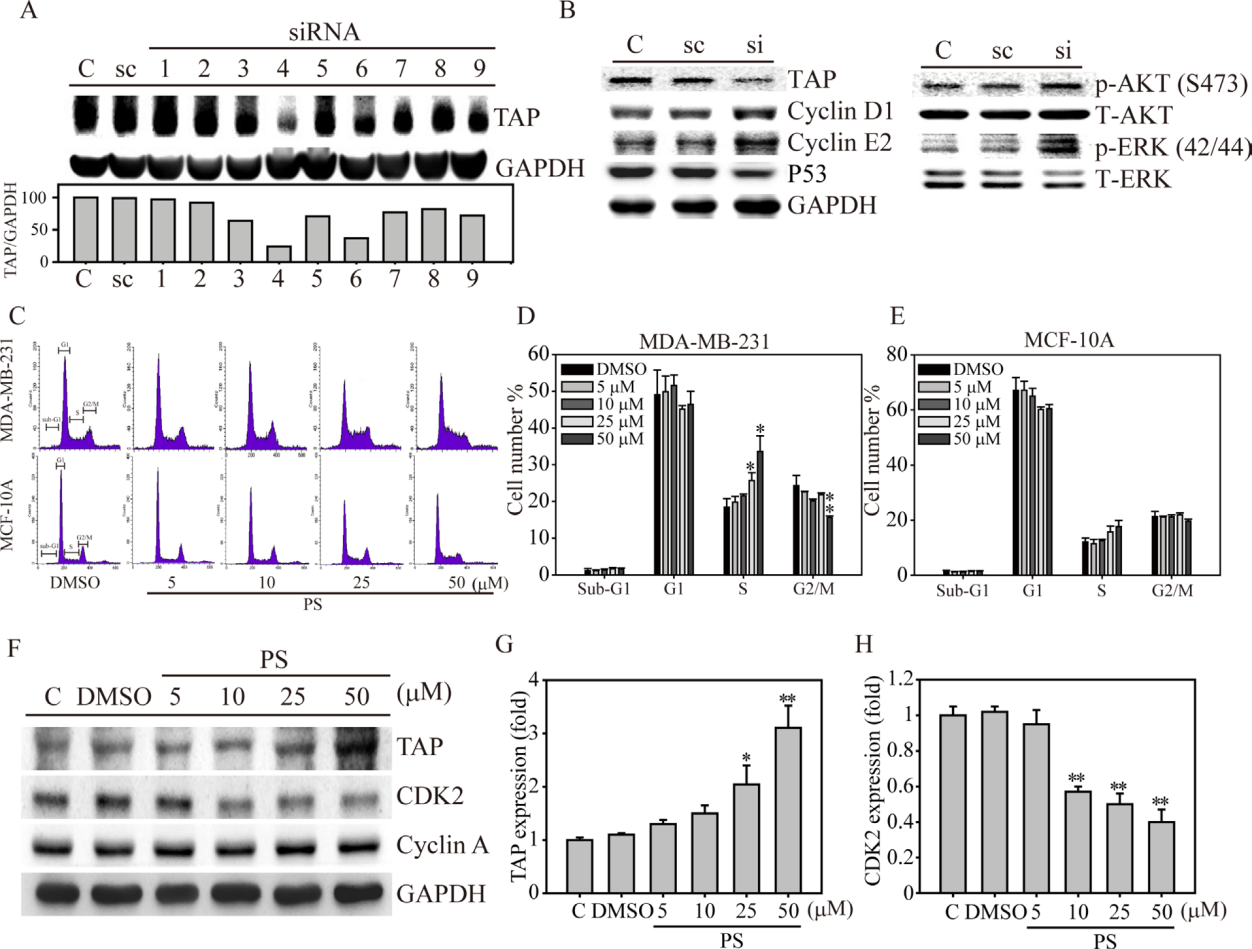
Role of TAP in cell cycle regulation in human breast cancer cells (**A**) MDA-MB-231 cells were transiently transfected with either sc or nine individual TAP siRNA clones for 24 hours. TAP protein expression was measured and quantified by density measurement. (**B**) sc (scramble), si (TAP siRNA 4), and C (MDA-MB-231) cells were analyzed for TAP, cell cycle protein (cyclin D1, E2 and P53), and signaling protein (AKT and ERK) expression. (**C**) Flow cytometry analysis of MDA-MB-231 and MCF-10A cells treated with 0–50 μM α-TOS for 24 hours. (**D**) Cell cycle phases for MDA-MB-231 (D) and MCF-10A (**E**) cells were measured and presented as cell population numbers. (**F**) MDA-MB-231 cells treated with PS (0–50 μM) in a dose-dependent manner were analyzed for TAP, CDK2, and cyclin A protein expression. Densitometry analysis of TAP (**G**) and CDK2 (**H**) protein expression is presented. All statistical tests were two-sided and compared to control. *P*-values less than 0.05 are indicated with an asterisk and those less than 0.01 are indicated with two asterisks.

To investigate whether TAP mediates downstream signaling and cell cycle regulation, we used nine individual TAP siRNA target sequences to knockdown TAP expression. As shown in Figure 3A, TAP siRNA sequence 4 yielded the greatest knockdown efficiency in MDA-MB-231 cells compared with that in the scramble (sc) and control cells. Evaluating the expression of cell cycle proteins (Figure 3B, left panel) showed that cyclin D1 and cyclin E2 were significantly induced in breast cancer cells in which TAP was knocked down. In contrast, the expression of the tumor suppressive protein P53 was significantly reduced in TAP knockdown breast cancer cells, indicating a strong correlation between TAP expression and cell cycle regulation. Meanwhile, proliferation signal transduction molecules, such as activated AKT and ERK, were induced in TAP knockdown cells, indicating the potential tumor-suppressive role of TAP in breast cancer cells via signal transduction inhibition and downstream cell cycle regulation.

According to previous findings [16], we hypothesized that the synergistic anti-cancer effects of PS and Vit. E together may coordinate TAP activity and downstream cell cycle regulation. We measured cell cycle distributions under dose-dependent PS treatment in both normal breast cells (MCF-10A) and breast cancer epithelial cells (MDA-MB-231). As shown in Figure 3C, increasing the concentration of PS from 5 to 50 μM gradually increased the length of the cell cycle S-phase in MDA-MB-231 cells, while the cycle of normal breast cells (MCF-10A) remained unchanged. The bar graph shown in Figure 3D depicting cell cycle analysis clearly demonstrates that the percentage of S-phase MDA-MB-231 cells was significantly induced from 19% with DMSO treatment to 26% and 33% when exposed to 25 and 50 μM PS, respectively. Meanwhile, the PS-enhanced S-phase duration also influenced the duration of the G2/M-phase in MDA-MB-231 cells, especially at the high PS concentration (50 μM). By contrast, the S-phase of the MCF-10A cell cycle was only slightly increased at 50 μM PS (Figure 3E). As expected, PS significantly induced TAP expression in MDA-MB-231 cells (Figure 3F). In addition, the S-phase cell cycle regulation of the CDK2 protein gradually decreased from 10–50 μM PS, whereas cyclin A was not affected during PS stimulation. To confirm this finding, we investigated the gene expression levels of TAP and CDK2 during PS exposure in breast cancer cells using Q-PCR. Consistently, 25 and 50 μM PS significantly induced TAP gene expression by 2.1- and 3.2-fold, respectively (Figure 3G), whereas CDK2 gene expression was remarkably reduced (Figure 3H). Notably, PS enhanced S-phase cell cycle arrest via TAP expression is also found in MCF-7 breast cancer cells, indicating that TAP enhanced cell cycle regulation may be not a cell-type specific phenomenon, at least not in breast cancer (Supplementary Figure 1A and 1B).

### α-TOS and PS synergistically inhibited signal transduction and enhanced breast cancer cell death

**Figure 4:**
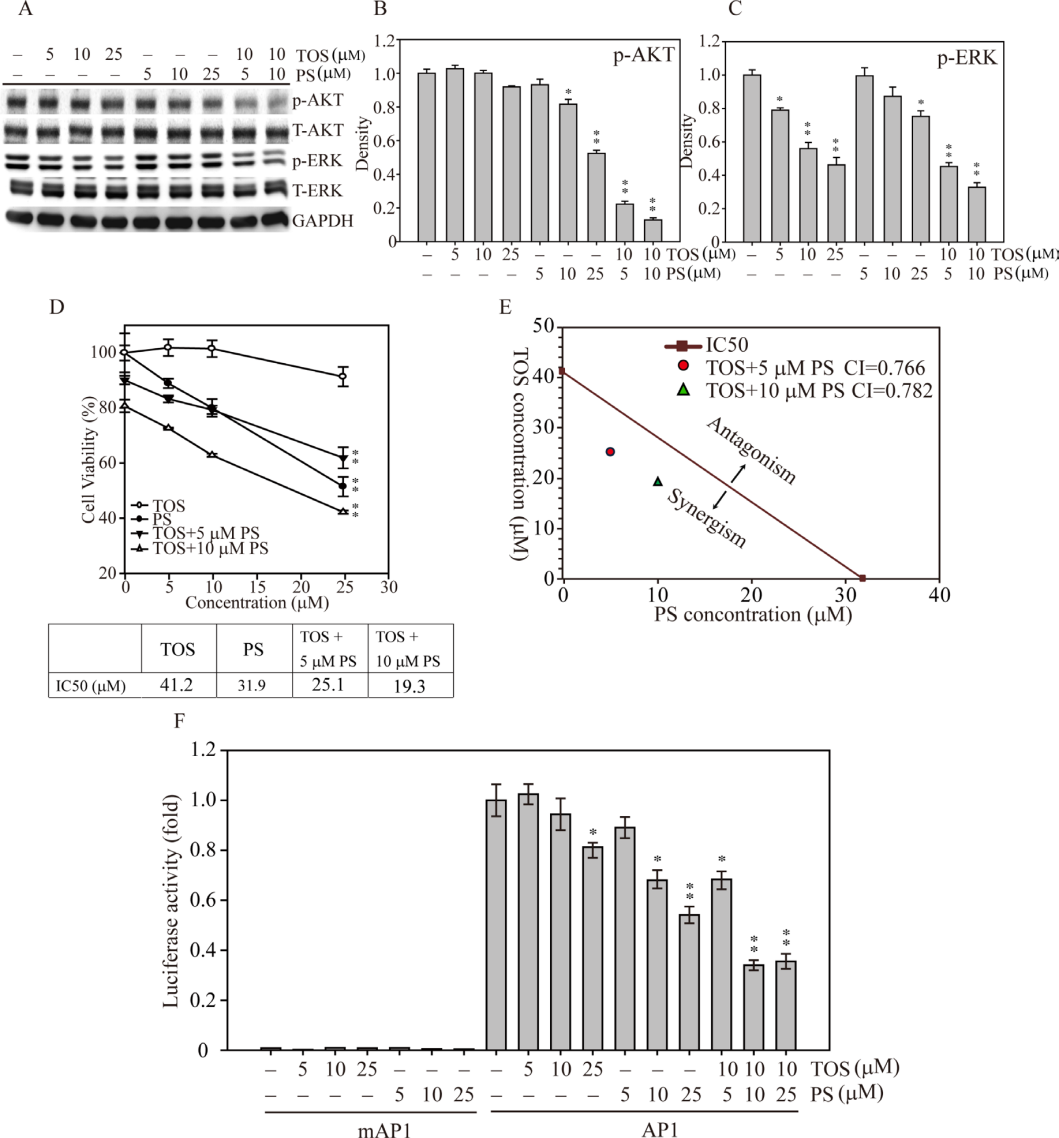
AKT and MAPK signaling-mediated cell growth regulation after α-TOS and PS treatment in MDA-MD-231 cells (**A**) Dose-dependent AKT and MAPK protein activation after treatment with α-TOS (0–25 μM) and PS (0–25 μM) alone or in combination. (**B**) Densitometry analysis of AKT and (**C**) ERK phosphorylated protein expression. (**D**) The IC50 of each drug treatment was calculated as the drug concentration able to reduce cell viability by 50%. (**E**) CI values of α-TOS and PS treatment on MDA-MB-231 IC50. The red dot and green triangle represent the synergistic effects of α-TOS treatment with 5 μM and 10 μM PS, respectively. (**F**) Luciferase activity of MDA-MB-231 cells transfected with either pGL3 (5′ AP1) or pGL3 (5′ mAP1) plasmids and treated with different concentrations of α-TOS (0–25 μM) or PS (0–25 μM) for an additional 12 hours. All statistical tests were two-sided and compared to non-treatment control. *P*-values less than 0.05 are indicated with an asterisk and those less than 0.01 are indicated with two asterisks.

To investigate whether both α-TOS and PS exposure could synergistically disrupt the signal transduction of MDA-MB-231 breast cancer cell growth, we treated the cells with increasing concentrations of two natural compounds in combination and by themselves. Figure 4A shows that AKT phosphorylation was significantly decreased at 10–25 μM PS, whereas AKT phosphorylation was unaffected by α-TOS alone. Interestingly, exposure to both α-TOS and PS significantly decreased ERK phosphorylation (α-TOS at 5, 10 and μM; PS at 25 μM) in breast cancer cells dose-dependently. In contrast, combinations of both compounds (10 μM α-TOS with 5 or 10 μM PS) exhibited more suppressive effects on the activation of both AKT and ERK compared with monotherapy treatment. Mild α-TOS treatment (10 μM) and a low concentration of PS (5 or 10 μM) largely inhibited AKT (Figure 4B) and ERK (Figure 4C) activation, suggesting that α-TOS and PS exposure in combination resulted in a highly effective synergistic disruption of breast cancer cell proliferation. To further explore the effects of α-TOS and PS monotherapy and combined therapy on IC50, we used the cell viability assay to measure MDA-MB-231 cell viability for 48 hours. Figure 4D shows that the IC50 cell viability measurements for α-TOS and PS treatments were 41.2 and 31.9 μM, respectively. However, with exposure to 5 and 10 μM PS in combination with α-TOS, the IC50 values for PS treatment significantly dropped to 25.1 and 19.3 μM, respectively.

To examine whether the α-TOS and PS combination was additive or synergistic, we used the approach designed by Chou and Talalay to calculate the combination index (CI) [21]. As shown in Figure 4E, the IC50 cell survival concentrations of MDA-MB-231 breast cancer cells were measured after both mono and combination drug exposure; the calculated CIs were less than one for the selected concentrations, demonstrating that the combination of both α-TOS and PS elicited a synergistic effect on cellular proliferation inhibition.

Next, to elucidate the downstream transcription factor regulatory effects of synergistic α-TOS and PS regulation of breast cancer cell proliferation, cell cycle progression, and apoptosis, we generated a luciferase-based AP1 activity assay to assess whether AP1 played a role in these cellular functions [22, 23]. Figure 4F shows that AP1 activity was significantly decreased to 70% and 60% of that of the control at 10 and 25 μM PS, respectively. In addition, combined with 10 μM α-TOS, the AP1 activity significantly decreased even further to 40% of the control with 10 or 25 μM PS co-exposure. Together, these results show that PS and α-TOS in combination significantly inhibit breast cancer cell growth via signal interruption and AP1 inactivation.

### PS and α-TOS synergistically inhibited breast tumor growth and metastasis in a xenograft tumor model

**Figure 5:**
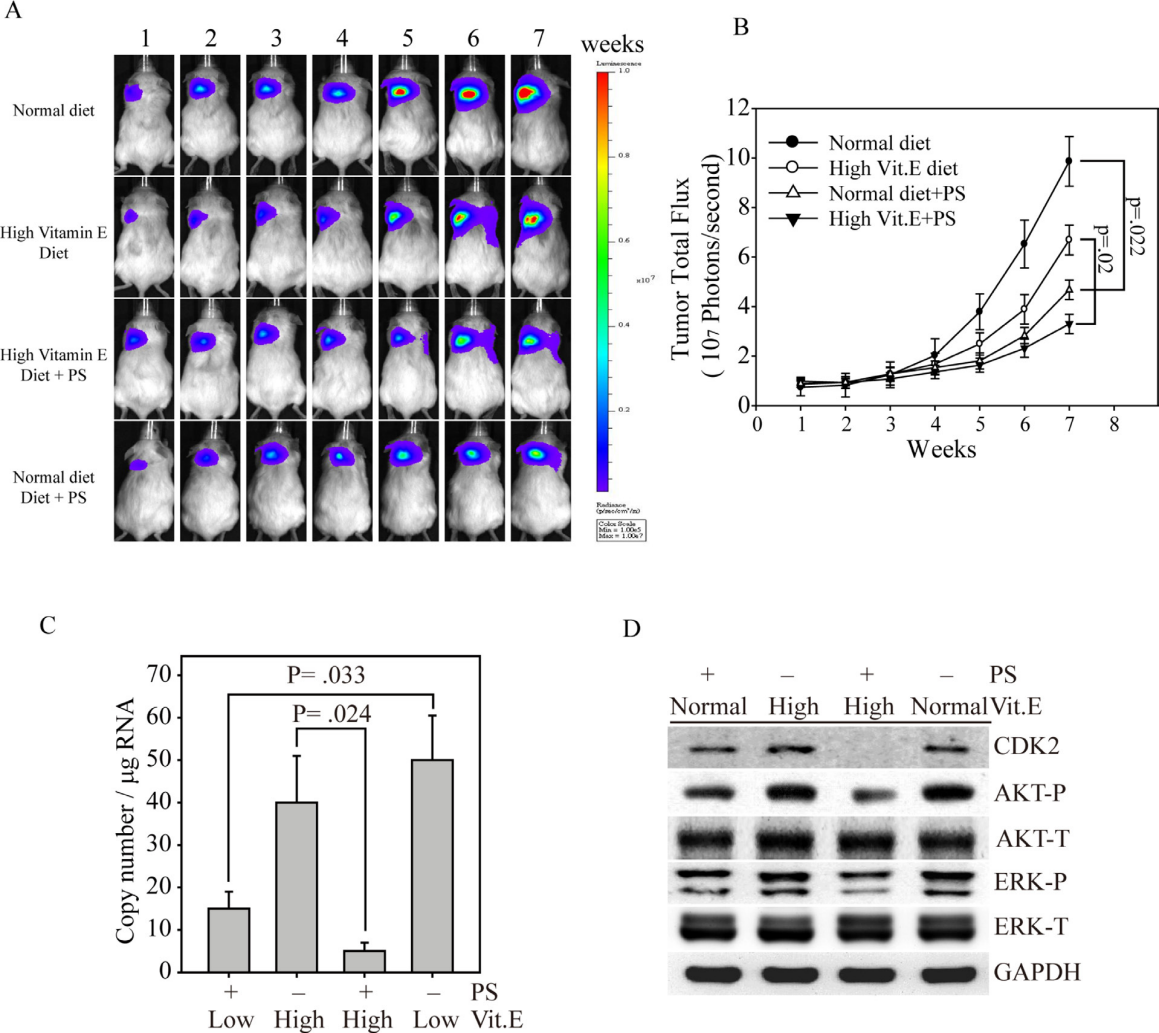
Synergistic effects of PS and α-TOS on tumor growth and metastasis in the MDA-MB-231 xenograft model (**A**) Bioluminescence imaging of tumor-bearing mice that were fed a high Vit. E diet, PS, or a combination of Vit. E and PS for seven weeks. (**B**) The photon flux for each mouse tumor was measured and calculated. (**C**) Before euthanasia, the blood from each mouse was harvested to measure circulating tumor cells by Q-PCR [17]. (**D**) At the end of the experiment, the mice were euthanized, and the expression levels of CDK2, AKT, and ERK in their tumor tissues were determined by western blotting. All statistical tests were two-sided and the actual *P*-value between groups is shown.

To investigate whether α-TOS and PS could synergistically inhibit cell growth *in vivo*, we generated MDA-MB-231 cells that stably expressed a bioluminescence gene (firefly luciferase), termed MDA-MB-231fluc2, for a xenograft breast tumor mouse model. After MDA-MB-231fluc2 cells were inoculated into SCID mice for three weeks, the xenograft-bearing mice were divided into four groups: normal diet, PS administration with normal diet, high Vit. E diet and PS administration with high Vit. E diet (Figure 5A). Mice fed the normal diet presented the highest breast cancer growth curve, followed by mice fed the high Vit. E diet, as determined by bioluminescence imaging (Figure 5B). In contrast, mice fed the high Vit. E diet with PS consistently exhibited the smallest tumor volume throughout the experiment. These data illustrate that PS alone exhibited great anti-breast cancer activity *in vivo* (black circle vs. empty triangle, *p* = .022). Furthermore, the combination of PS and α-TOS *in vivo* might be a better breast cancer therapeutic strategy (empty circle vs. reverse triangle, *p* = 0.02). To determine whether the PS and α-TOS combination could stop cancer metastasis, we used *our recently developed* novel technique to evaluate CTCs from the xenograft-bearing mice [17]. Figure 5C shows that the CTC numbers were significantly inhibited in mice that were fed the high Vit. E diet and treated with PS compared with mice that were only fed the high Vit. E diet (*p* = 0.024). In contrast, mice that were fed the normal diet and administered PS also exhibited a CTC inhibition ability compared with mice that were fed the normal diet (*p* = 0.033). Figure 5D shows that the tumors from mice that were fed the high Vit. E diet with PS administration presented the lowest CDK2, phospho-AKT, and phospho-ERK expression levels compared with those of the other tumor groups. Interestingly, all of the tumors from mice that received PS maintained low expression levels of these cell proliferation proteins. These results indicate that the PS inhibitory effects on tumor growth and metastasis are closely associated with certain signaling pathways, such as the AKT and MAPK pathways.

### The mechanism of the synergistic anti-tumor effect of PS and α-TOS on breast cancer

**Figure 6:**
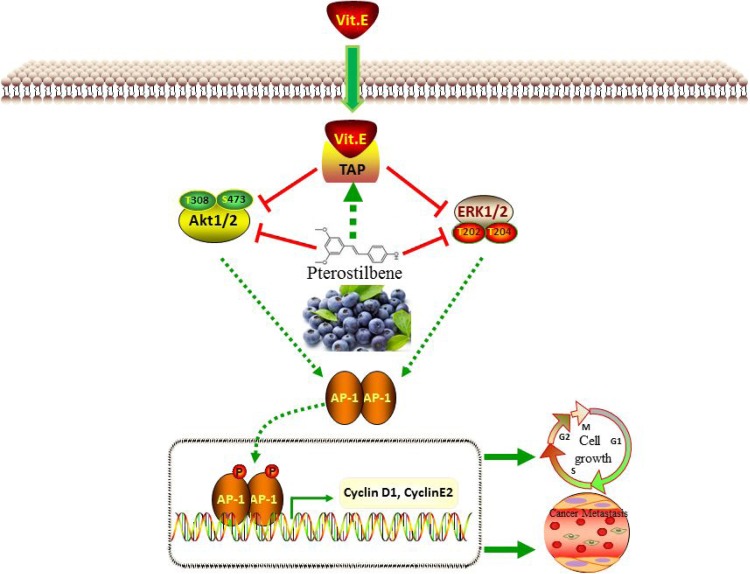
Schematic representation of the mechanism of the synergistic anti-tumor effects of PS and α-TOS in breast cancer

In conclusion, this study aimed to evaluate the potential anti-tumor benefits of α-TOS and PS in breast cancer cells *in vitro* and *in vivo* (Figure 6). The results suggest that co-treatment of α-TOS and PS largely improved cancer cell growth inhibition through TAP activation. These results were associated with the downregulation of AKT and ERK activation and triggered downstream transcription factor and cell cycle protein regulation. In an animal model, the synergic inhibition effects on both tumor growth and cancer metastasis were also shown.

## DISCUSSION

The relationship between Vit. E and cancer risks has been investigated in many epidemiologic studies [24]. Two large randomized and controlled clinical trials failed to meet expectations for the prevention of prostate cancer [25]. Although these findings were clearly disappointing, they showed that α-TOS might have greater health benefits. Recently, a study demonstrated that dietary Vit. E and Vit. C inflict dose-dependent protective effects on both colon and rectal cancer [26], and another clinical study showed a preventative effect of Vit. E and Vit. C in prostate cancer [27]. Among 29,000 elderly male smokers, a lower number of prostate cancer diagnoses (32% less) was detected in patients taking Vit. E for six years or longer; prostate cancer deaths were also reduced in this group (41% less). Based on clinical observations, Vit. E has also been suggested to have a protective role against lung cancer, specifically against cigarette smoking-induced lung cancer [24]. These studies indicate the variety of Vit. E anti-cancer effects that could be applied to different cancer treatments.

Meanwhile, Fulan and colleagues found that a sufficient amount of dietary Vit. E and total Vit. E consumption significantly reduced breast cancer risks by 18% [28]. Other studies have also illustrated an inverse association between Vit. E intake and breast cancer risk in India, Finland, Uruguay, Italy, and the U.S. [29–34]. Among these studies, breast cancer risks were significantly higher in patients with α-tocopherol intake lower than 6 mg/day or serum α-tocopherol concentrations lower than 25 μM/L compared with those in patients with α-tocopherol intake higher than 9.7-13 mg/day or serum α-tocopherol concentrations greater than 38 μM/L. These data clearly illustrate that the potential anti-breast cancer activity of α-tocopherol may be strongly associated with its serum levels.

Lately, berries have garnered increasing attention for their chemopreventative and therapeutic potential against breast cancer *in vitro* and *in vivo* [35]. In a recent study, blueberry juice extract exhibited anti-proliferative effects against TNBC cells, including HCC38, HCC1937, and MDA-MB-231 cells, without affecting the proliferation of normal breast epithelial cells (MCF-10A) [36]. Treatment with blueberry extract significantly decreased signaling transduction of the PI3K/AKT pathway and resulted in the inhibition of breast carcinogenesis. Similar to blueberry extract, in the current study, PS exposure inhibited carcinogenic pathways in breast cancer cells (MDA-MB-231) but not in normal breast epithelial cells (MCF-10A), indicating that PS may be the primary agent responsible for the anti-carcinogenic effects of blueberry extract in several malignancies, especially breast cancer [37]. The daily amount of PS consumption can vary according to fruit intake, and the PS content per blueberry has been estimated to vary from 99 to 520 ng/g depending on the type of berry selected [38].

In a previous study, α-TOS demonstrated strong cell cycle arrest at the S-phase in MDA-MB-453 breast cancer cells following MEK and ERK kinase inhibition and the upregulation of the tumor suppressive protein P21 [39]. In addition, cell cycle and protein analyses indicated that the cell cycle arrest was due to α-TOS-induced P53 activation and reduced expression of E2F, which is a key regulatory transcription factor in the cell cycle checkpoint [40]. The anticancer properties of Vit. E with chemotherapeutic agents were also evaluated in several cancer types. One study showed that α-TOS increases the levels of apoptosis induced by TRAIL through Caspase- and p53-dependent activation in colon cancer cells both *in vitro* and *in vivo* [5]. In addition to enhancing the apoptotic effects of the combined α-TOS and TRAIL treatment, α-TOS has been shown to increase the growth inhibitory effects of cisplatin, tamoxifen and decaprazine in melanoma cells [41] and parotid acinar carcinoma cells [42], as well as those of adriamycin in prostate carcinoma cells [43] and those of doxorubicin in leukemia cells [44]. In this study, we found that cell cycle protein regulation was strongly impacted by α-TOS exposure in MDA-MB-231 cells using microarray and protein analyses. TAP mediates P53 activation, and downstream signaling regulation may initiate Vit. E-induced cell cycle arrest and cell apoptosis.

Metastasis refers to the spread of cancer to various parts of the body, typically to bones, liver, lung, and brain. According to a report from the National Cancer Institute, an estimated 155,000 Americans are currently living with metastatic breast cancer, resulting in more than 40,000 deaths annually in the U.S. [45]. However, patients do not die from breast cancer that remains in the breast, and metastasis occurs when cancerous cells travel to vital organs, threatening life. To achieve the maximum anti-tumor effect from available Vit. E analogs, a natural compound, PS, was investigated for its synergistic pharmacological effects. In summary, this study shows that α-TOS and PS synergistically suppressed breast cancer cell proliferation and tumor growth *in vitro* and *in vivo* through the inhibition of downstream AP1 activation and cell cycle protein regulation. In addition, our recently developed CTC detection assay was used to show that the combination of α-TOS and PS largely suppressed the invasion ability of MDA-MB-231 cells in a xenograft animal model. These results suggest that a combination of Vit. E and PS as dietary supplements may be beneficial during breast cancer treatment.

## MATERIALS AND METHODS

### Cell culture

A human mammary gland epithelial adenocarcinoma cell line (MDA-MB-231) was purchased from American Type Culture Collection (ATCC, Manassas, VA, USA). The cells were maintained in complete culture medium composed of an equal mixture of Dulbecco's Modified Eagle's Medium (DMEM) and Ham's F12 medium. Cells were grown in a 37°C humidified incubator with 5.0% CO_2_ and supplemented with 10% (v/v) fetal bovine serum (FBS, Biological Industries Co., Haemek, Israel), 100 units/mL penicillin, and 100 mg/mL streptomycin.

### Cell proliferation assay

Cell growth was determined using the 3-(4,5- dimethylthiazol-2-yl)-2,5-diphenyltetrazolium (MTT) assay [46]. In 96-well plates, 3,000 cells were seeded and exposed to PS or α-TOS according to the experimental protocol. After 48 hours of treatment, the medium was replaced with fresh medium containing 1 μg/mL MTT for two hours. Dimethyl sulfoxide (DMSO) was added, and the absorbance at 570 and 630 nM was determined.

### Chemicals and reagents

α-TOS was purchased from Sigma-Aldrich Chemical Co. (St Louis, MO, USA). PS (96% purity) was a gift from Prof. Chi-Tang Ho. The chemicals used in this study were dissolved in dimethyl sulfoxide (DMSO).

### RNA interference

Nine human TAP siRNA and TAP scramble (sc) sequences were cloned into a pSuperior vector using *Bgl*II and *Hin*dIII restriction enzymes. The target sequences of TAP siRNAs 1 through 9 and TAP sc are presented in Supplementary Table 1. The construct was confirmed by DNA sequence analysis, and the transfection protocol is described below.

### Transfection and cell line selection

MDA-MB-231 cells were transfected with pcDNA3 plasmids expressing the firefly luciferase gene (the gene sequences were originally from *luc4.1*; Chris Contag, Stanford University, Stanford, CA, USA) by electroporation as described previously [47]. Briefly, 5 × 10^6^ cells were washed twice with PBS and mixed with 10 mg of the plasmid. Two pulses were applied for 20 milliseconds under 1.2 kV using the pipette-type MicroPorator MP-100 (Digital Bio, Seoul, Korea). Stable cells were selected 48 hours later with G418 (6 mg/mL). MDA-MB-231 bioluminescent derivatives were used for subsequent *in vivo* studies.

### Activator protein 1 (AP1) activity assay

The 5× AP1 and 5× mutant AP1 binding promoter-luciferase gene fusion constructs were obtained using the pGL3-Basic vector (Promega, Madison, WI, USA). Briefly, suitable 5× AP1 and 5× mutant AP1 binding sequences were introduced into the polylinker upstream from the luciferase gene. These constructs are referred to as 5× AP1-pGL3 and 5× mAP1-pGL3. These fragments were generated with restriction enzymes and cloned directly into the pGL3-Basic vector. Five million MDA-MB-231 cells were transiently co-transfected with 9 μg of 5× AP1-pGL3 or 5× mAP1-pGL3 and 1 μg of the RLTK plasmid (Promega) using the MicroPorator MP-100 (Digital Bio) according to the manufacturer's instructions. After 24 hours of incubation, the medium was replaced by culture medium supplemented with 10% FBS and different concentrations of PS or α-TOS. After 24 hours of drug exposure, the cells were lysed with reporter lysis buffer (Promega), and luciferase activity was determined by the Hidex Chameleon microplate reader (Turku, Finland) using luciferase assay reagent (Promega). Relative luciferase units were normalized to that of Renilla luciferase from the same cell lysates. Each luciferase assay was performed at least three times.

### Protein extraction, western blotting, and antibodies

For western blot analysis, MDA-MB-231 cells transfected with TAP siRNAs 1 through 9 and sc were washed once with ice-cold PBS and lysed with radioimmunoprecipitation assay (RIPA) lysis buffer containing protease inhibitors as previously described [48]. For *in vivo* experiments, tumor tissues were cut into small pieces and lysed with RIPA lysis buffer containing protease inhibitors. Cell and tumor samples were homogenized three times at setting 3 (18,000 rpm) on ice using a PRO 200 homogenizer (PRO Scientific Inc., Monroe, CT, USA). Fifty micrograms of protein from each sample was resolved by SDS/PAGE and transferred to nitrocellulose membranes. Antibodies were purchased from the following vendors: anti-AKT, anti-ERK, anti-phospho ERK, and anti-GAPDH from Santa Cruz Biotechnology (Santa Cruz, CA, USA); anti-TAP, anti-Cyclin D1, anti-Cyclin E2, anti-Cyclin A, anti-CDK2 and anti-P53 from Abcam (Cambridge, UK); and anti-phospho AKT (Ser473) from Cell Signaling Technology (Danvers, MA, USA). Secondary anti-mouse and anti-rabbit antibodies were purchased from Santa Cruz Biotechnology. The anti-GAPDH and anti-TAP primary antibodies were used at dilutions of 1:8,000 and 1:1,00, respectively, for membrane hybridization for two hours, followed by a one-hour incubation with the appropriate secondary antibody at a 1:4,000 dilution. GAPDH expression served as the control for all western blot assays, which were repeated at least twice.

### Combination index (CI)

The CI is a mathematical and quantitative representation of a two-drug pharmacological interaction. Using data from the growth inhibition experiments and computerized software, CI values were generated over a range of α-TOS and PS treatments. The detailed calculation was originally described by Chou and Talalay [21]. CI = 1 indicates an additive effect, CI < 1 indicates a synergistic effect, and CI > 1 indicates an antagonistic effect.

### Blood sample collection

In total, 100–150 μl of blood from each mouse was obtained by cardiac puncture and processed according to standard separation protocols. Total DNA was isolated from human cell lines and mouse leukocytes using the AxyPrep blood genomic DNA miniprep kit following the manufacturer's protocol. A NanoDrop spectrophotometer was used for DNA quantitation (260/280) measurements, and all DNA samples contained at least 10 ng/μl DNA.

### Real-time quantitative polymerase chain reaction (Q-PCR)

The human cell cycle-related gene primers are listed in a Supplementary Table 1. All oligo primers were synthesized by Genomics BioSci and Tech (Taipei, Taiwan). A LightCycler thermocycler (Roche Molecular Biochemicals, Mannheim, Germany) was used for Q-PCR analysis. One microliter of the sample and master mix was first denatured for 10 minutes at 95°C and then incubated for 40 cycles (denaturation at 95°C for 5 seconds, annealing at 60°C for 5 seconds, elongation at 72°C for 10 seconds) to detect fluorescence intensity. All PCR samples underwent melting curve analysis for the detection of non-specific PCR products. Q-PCR gene expression levels were normalized with those of human β-glucuronidase (GUS) as the internal control using built-in Roche LightCycler Software Version 4.

### DAVID functional annotation analysis

The DAVID [20] was used for the gene annotation of microarray data. Analysis of the DAVID functional annotation tool was run online (https://david.ncifcrf.gov/) using default parameters while focusing on the categories Gene Ontology-Molecular Function and Gene-Ontology-Biological Process. Similarities among annotation terms are listed in combination with a heatmap indicating the top 26 genes that were up- and down-regulated by α-TOS exposure and functionally clustered into common gene ontology (GO) terms (Supplementary Table 2).

### Flow cytometry

MDA-MB-231 and MCF-10A cells were plated at a density of 5 × 10^6^ cells in 10-cm Petri dishes prior to the experiment. PS was added at final concentrations of 5, 10 25 and 50 μM and incubated for 24 hours. The cells were then washed with PBS and fixed gently with 70% ethanol in a freezer for 2 h before being treated with 0.25% Triton X-100 for 5 min in an ice bath. Cells were resuspended in 1 ml of PBS containing 40 μg/mL propidium iodide (PI) and 0.1 mg/mL RNase. The cells were incubated in a dark room for 20 min at room temperature, and cell cycle analysis was performed using a FACScan flow cytometer (Becton Dickinson, Mountain View, CA, USA) and FlowJo 9.0 software (Tree Star, Ashland, OR, USA). For each measurement, at least 10,000 cells were counted.

### Animal experiments

Four-week-old severe combined immunodeficient (SCID) female mice were purchased from the National Science Council Animal Center (Taipei, Taiwan) and housed in microisolator cages at the Laboratory Animal Center of Taipei Medical University (Taipei, Taiwan). For the human cancer xenograft studies, 5 × 10^6^ viable MDA-MB-231 breast cancer cells were injected subcutaneously into the right mammary fat pads of SCID mice. After tumor establishment, the mice were divided into four groups (five mice in each group): normal diet (Vit. E, 42 IU/kg), high Vit. E diet (99 IU/kg), PS (40 μg/kg) with normal diet, and PS with high Vit. E diet. PS was administered orally three times per week, and tumor size was measured using bioluminescence imaging every week.

### Bioluminescence imaging

Bioluminescence imaging was performed with a highly sensitive cooled charge-coupled device (CCD) camera mounted in a light-tight specimen box (*In Vivo* Imaging System, Xenogen, Alameda, CA, USA) according to the manufacturer's instructions. Briefly, the mice were administered D-luciferin (50 mg/kg) in PBS by i.p. injection and anesthetized (2.5% isoflurane). Luciferase activity in the tumors was displayed and quantified as total photons per second using Living Image**^®^** software (Xenogen). On day 60, the tumors were excised, and the protein expression levels of CDK2, AKT-P, AKT-total, ERK-P, ERK-total and GAPDH were determined by western blotting.

### Statistical methods

Significant differences in activator protein 1 (AP1) luciferase activity, protein expression, bioluminescence imaging, and cell proliferation were analyzed by Student's *t*-tests for comparing significance between groups. All statistical comparisons were performed using SigmaPlot graphing software (San Jose, CA, USA). *P*-values less than 0.05 are indicated with an asterisk, and those less than 0.01 are indicated with two asterisks; all statistical tests were two-sided.

## SUPPLEMENTARY MATERIALS FIGURE AND TABLES



## Abbreviations

Vit. E: Vitamin E
α-TP: α-tocopherol
TNBC: triple negative breast cancer
TAP: tocopherol-associated protein
α-TOS: α-tocopheryl succinate
Res: resveratrol
PS: pterostilbene (3,5-dimethoxi-49-hydroxystilbene)
DAVID: Database for Annotation, Visualization and Integrated Discovery
GO: gene ontology
Q-PCR: real-time quantitative polymerase chain reaction
CI: combination index
CTC: circulating tumor cell
SC: scramble
SCID: severe combined immunodeficient
IVIS: *In Vivo* Imaging System
AP1: activator protein 1
