# Supplementary material for: α-Tocopherol succinate enhances pterostilbene anti-tumor activity in human breast cancer cells in vivo and in vitro
Source: Oncotarget. 2017 Dec 17;9(4):4593–606. doi: 10.18632/oncotarget.23390 (PMC5796998; doi:10.18632/oncotarget.23390)
Supplement: Supplementary file 1 [file oncotarget-09-4593-s001.pdf]

α-Tocopherol succinate enhances pterostilbene anti-tumor activity in human breast cancer cells *in vivo* and *in vitro*

SUPPLEMENTARY MATERIALS

Supplementary Table 1: The RNAi sequences targeting TAP and the real-time PCR primer sequences for both the human and mouse *GUS* genes

TAP RNAi	Targeting sequence
TAP 1	AAACTTGGCCAATGCCTCC
TAP 2	ATAGTCATCTGGATTCGGC
TAP 3	TTATGTCGTACCAGACTGG
TAP 4	AACTTTGTGGTCTGGTGGG
TAP 5	TATGGTGATGGTCTCCACC
TAP 6	AAACTCTCCATAGGCCTCC
TAP 7	TTTGATGAGGTTATAGGCC
TAP 8	ATACTGCTGTTTCACCTGG
TAP 9	TAGATGCCAGGATCACTGC
TAP sc	GGGAACAAACAAGCACGAA
**Realtime PCR**	**Primer sequence**
Human Cyclin E2 sense	AGGAAACATTCATTCAAATAGCTC
Human Cyclin E2 anti sense	GTCCCACTCCAAACCTG
Human Cyclin D1 sense	ATGTTCGTGGCCTCTAAG
Human Cyclin D1anti sense	AGGAAGTGTTCAATGAAATCG
Human p53 sense	CTGGAAGACTCCAGTGGTA
Human p53 anti sense	TTGGACAGTGCTCGCTTA
Human TAP sense	TGGAGCGGATGTTGGTTT
Human TAP anti sense	TTGGCATGAATGAAGCTGTAGG
Human CDK2 sense	TCCAGGGCCTAGCTTTC
Human CDK2 anti sense	CCTCATGGGTGTAAGTACGA
Human GUS sense	CCTGTTTACTTGAGCAAGACTGATA
Human GUS anti sense	CCTTTAGTGTTCCCTGCTAGAATA
Mouse GUS sense	TGAACTCTTGAAAGCCTGC
Mouse GUS anti sense	GAAATGGAGGACCAGCTCATA

Supplementary Table 2: The list of the top 26 genes that were up- and down-regulated by α-TOS exposure and functionally clustered into common GO terms

TargetID	ProbeID	DMSO.AVG_Signal	α-TOS.AVG_Signal	Fold change
**ABCG1**	5860377	364.7464	242.1847	0.663981056
**ZFP91**	4490114	2780.348	1877.219	0.675174115
**TMED7**	6560162	582.8218	409.086	0.701905797
**CCND1**	5820601	2328.695	1649.236523	0.7082235
**ABCB10**	4810048	300.6488	215.0966	0.715441405
**ORC3L**	6770692	411.9026	297.3176	0.721815303
**IL10**	3990703	2616.901	1912.835	0.730954285
**CCNE2**	4760154	742.9545	560.3895	0.754271628
**GPR1**	3870386	646.459	482.0336	0.745652238
**TTK**	5870725	497.9786	370.9729	0.744957514
**CCNE1**	2070131	194.5154	215.9883	1.110391774
**EI24**	770541	2877.802	3356.19	1.166233813
**DUSP6**	6250468	267.559	334.7671	1.251189831
**TCEB1**	5050242	1414.811	1800.19	1.272389033
**PTPN5**	3180170	108.4975	138.5369	1.276867209
**CDC25B**	4760338	682.0165	873.0212	1.280058767
**PSMD4**	6110446	816.3845	1054.151	1.291243281
**UBE2Z**	4860458	887.3458	1148.032	1.293781973
**BAIAP2**	6590050	130.8824	169.81	1.297424253
**BAD**	5860376	687.7907	894.6017	1.300688858
**TP53**	3710543	111.1629	146.3971	1.316960065
**MPZL1**	2490470	211.0256	289.9285	1.373902029
**PVRL1**	1740592	253.6305	350.2217	1.380834324
**CLDN7**	5080373	1766.844	2457.459	1.390874916
**IGFBP3**	6590132	904.8586	1279.995	1.414580134
**ULK2**	1030327	130.869	198.4758	1.516599042

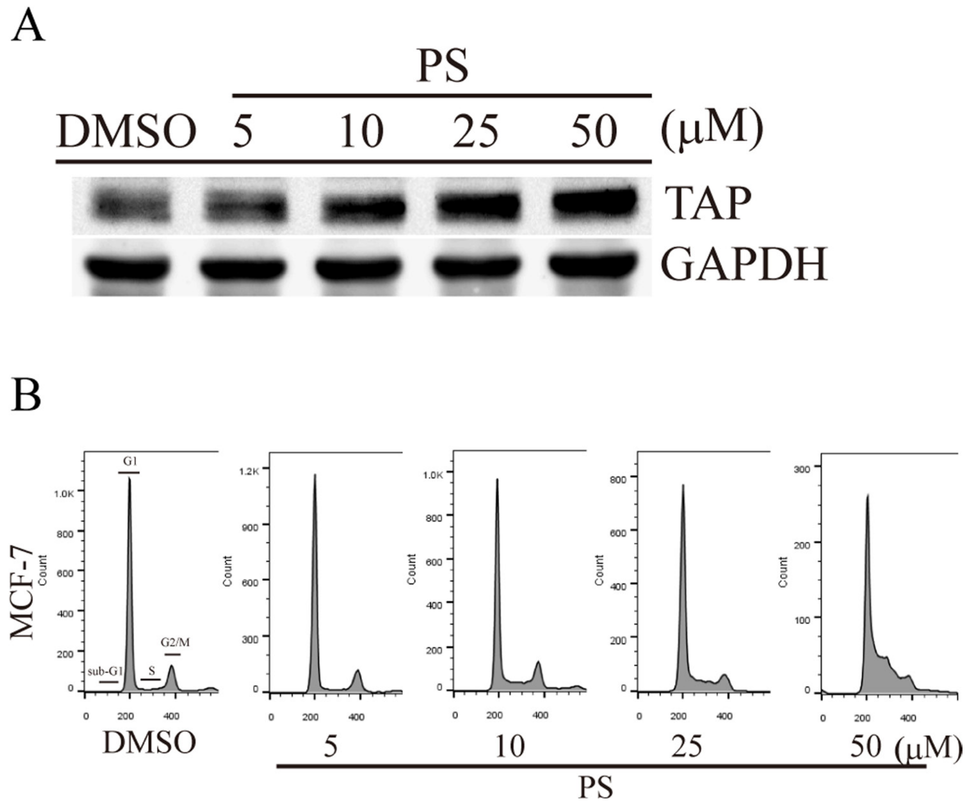

Supplementary Figure 1: Western blotting of TAP expression (**A**) and flow cytometry analysis (**B**) of MCF-7 cells treated with 0–50 μM α-TOS for 24 hours.
